# Optimizing LI-RADS: ancillary features screened from LR-3/4 categories can improve the diagnosis of HCC on MRI

**DOI:** 10.1186/s12876-024-03201-2

**Published:** 2024-03-21

**Authors:** Zi-xin Zhang, Hui Xv, Yan-ni Du, Zhi-bin Lv, Zheng-han Yang

**Affiliations:** 1 Department of Radiology, Beijing Friendship Hospital, Capital Medical University, Beijing, China; 2 Department of Radiology, Beijing Ditan Hospital, Capital Medical University, Beijing, China

**Keywords:** Hepatocellular carcinoma, LI-RADS, MRI, Ancillary feature

## Abstract

**Objective:**

To determine the high-efficiency ancillary features (AFs) screened from LR-3/4 lesions and the HCC/non-HCC group and the diagnostic performance of LR3/4 observations.

**Materials and methods:**

We retrospectively analyzed a total of 460 patients (with 473 nodules) classified into LR-3-LR-5 categories, including 311 cases of hepatocellular carcinoma (HCC), 6 cases of non-HCC malignant tumors, and 156 cases of benign lesions. Two faculty abdominal radiologists with experience in hepatic imaging reviewed and recorded the major features (MFs) and AFs of the Liver Imaging Reporting and Data System (LI-RADS). The frequency of the features and diagnostic performance were calculated with a logistic regression model. After applying the above AFs to LR-3/LR-4 observations, the sensitivity and specificity for HCC were compared.

**Results:**

The average age of all patients was 54.24 ± 11.32 years, and the biochemical indicators ALT (*P* = 0.044), TBIL (*P* = 0.000), PLT (*P* = 0.004), AFP (*P* = 0.000) and Child‒Pugh class were significantly higher in the HCC group. MFs, mild-moderate T2 hyperintensity, restricted diffusion and AFs favoring HCC in addition to nodule-in-nodule appearance were common in the HCC group and LR-5 category. AFs screened from the HCC/non-HCC group (AF-HCC) were mild–moderate T2 hyperintensity, restricted diffusion, TP hypointensity, marked T2 hyperintensity and HBP isointensity (*P* = 0.005, < 0.001, = 0. 032, *p* < 0.001, = 0.013), and the AFs screened from LR-3/4 lesions (AF-LR) were restricted diffusion, mosaic architecture, fat in mass, marked T2 hyperintensity and HBP isointensity (*P* < 0.001, = 0.020, = 0.036, < 0.001, = 0.016), which were not exactly the same. After applying AF-HCC and AF-LR to LR-3 and LR-4 observations in HCC group and Non-HCC group, After the above grades changed, the diagnostic sensitivity for HCC were 84.96% using AF-HCC and 85.71% using AF-LR, the specificity were 89.26% using AF-HCC and 90.60% using AF-LR, which made a significant difference (*P* = 0.000). And the kappa value for the two methods of AF-HCC and AF–LR were 0.695, reaching a substantial agreement.

**Conclusion:**

When adjusting for LR-3/LR-4 lesions, the screened AFs with high diagnostic ability can be used to optimize LI-RADS *v*2018; among them, AF-LR is recommended for better diagnostic capabilities.

**Supplementary Information:**

The online version contains supplementary material available at 10.1186/s12876-024-03201-2.

## Introduction

Hepatocellular carcinoma (HCC) is the most common primary malignant tumor of the liver, ranking fifth in incidence and second in mortality worldwide [1, 2]. Among them, approximately 80% of hepatocellular carcinomas are related to chronic hepatitis B virus (HBV) or hepatitis C virus (HCV) infection, which are also the main high-risk factors for the incidence of hepatocellular carcinoma in China. Early diagnosis and timely treatment can make the 5-year survival rate of HCC reach 50-70%. However, the prognosis of advanced HCC with delayed diagnosis is poor [3, 4]. For HCC diagnosis, imaging plays a critical role, especially magnetic resonance imaging (MRI), which is widely used for its high soft tissue resolution and multisequence scanning [5].

Based on this, the Liver Imaging Reporting and Data System (LI-RADS) was launched in 2011 and was most recently updated in 2018 to standardize liver imaging manifestations, and the interpretation and reporting system targeted high-risk patients for HCC, such as those with cirrhosis, chronic HBV infection without cirrhosis, or current or prior HCC, including adult liver transplant candidates and recipients [6–8]. Each category reflects the probability of benignity, malignancy, and HCC. The LR-3 (intermediate probability of malignancy), LR-4 (probable HCC), and LR-5 (definite HCC) categories are assigned based on a combination of major features (MFs) and ancillary features (AFs). The treatment methods and follow-up procedures are different for different categories of lesions. LR-5 lesions provide nearly 100% specificity for the diagnosis of HCC, and active surgical resection or intervention is necessary [9, 10]. However, for lesions with insufficient number or insufficient combination of main features of observed HCC lesions, the corresponding classification will be LR-4 or LR-3. Although other guidelines consider the vast majority of observations in the LR-4 category to be HCC [11–14], multidisciplinary discussion is still needed for LR-4 lesions to determine whether immediate treatment or regular follow-up observation is needed. The LR-3 category indicates an intermediate probability of malignancy, and regular imaging follow-up is recommended rather than active treatment [3, 4]. Given that the treatment strategies for these two types are completely different, improving the correct classification of LR-3 or LR-4 types is of clinical importance.

At the same time, although AFs can be selectively used for classification adjustment, improve detection and increase confidence, the number of AFs is as high as 21, and most of the features appear less frequently [15], taking into account that both MFs and AFs make the LI-RADS system more complex and significantly increase the workload of radiologists. Therefore, in this study, we tried to screen different combinations of high diagnostic efficiency AFs to adjust the categories of LR-3 and LR-4 lesions, hoping to improve the diagnostic ability of the above two types of lesions.

## Materials and methods

### Patients

**Fig. 1:**
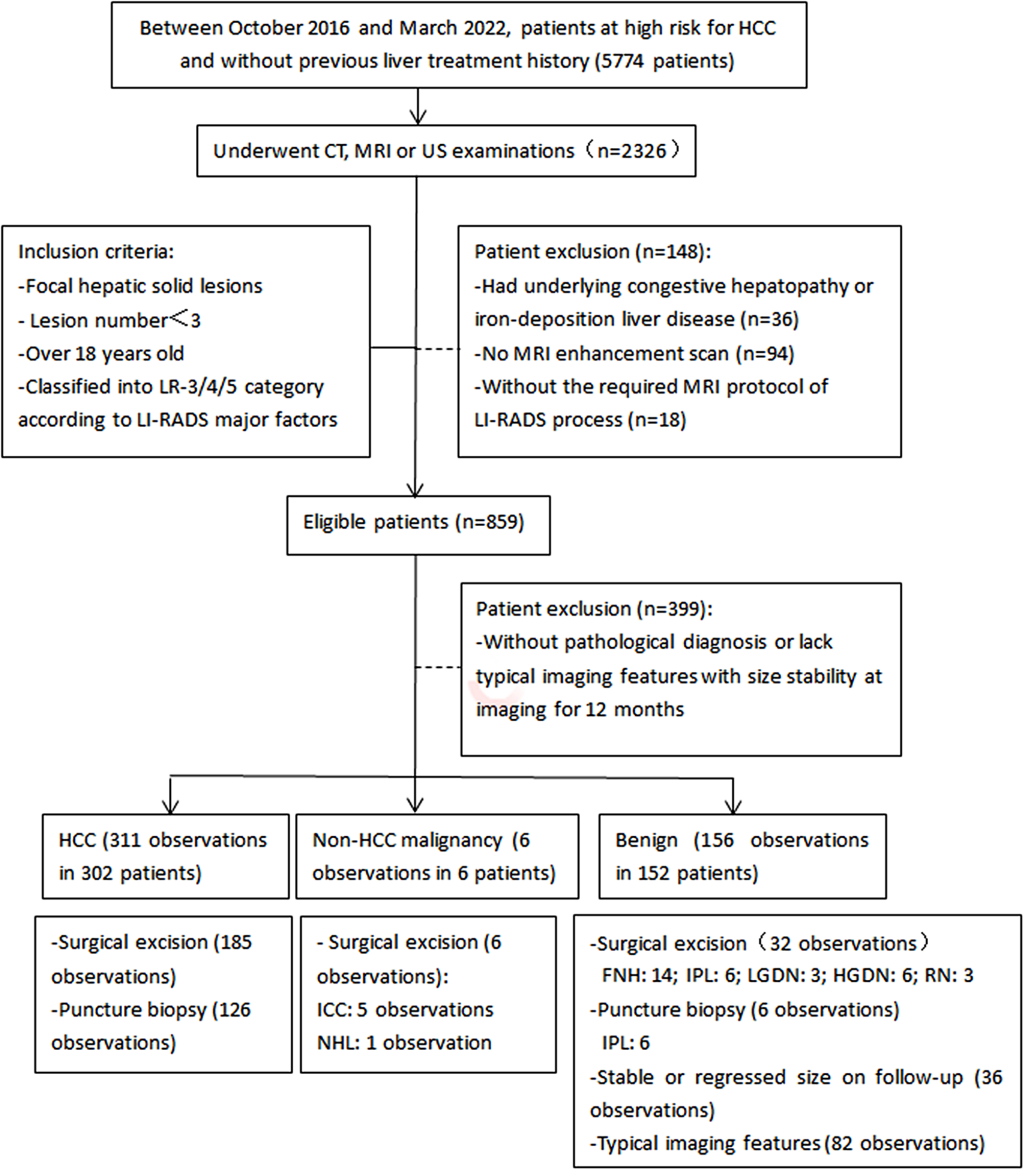
Flowchart of study population

The single-center study was approved by the Ethics Review Committee of Beijing Friendship Hospital, Capital Medical University, and written informed consent from the patients for use of data was waived due to the retrospective nature of the study. The following medical record data were collected from October 2016 to March 2022 on patients at high risk for HCC and without previous liver treatment history: age, sex, clinical features, laboratory indicators, MRI findings and follow-up for 12 months. A total of 460 patients with 473 lesions were enrolled. The inclusion criteria were as follows: (1) patients with focal hepatic solid lesions; (2) the number of the lesions was fewer than 3; (3) patients was older than 18 years old; and (4) lesions classified into LR-3/LR-4/LR-5 categories according to LI-RADS MFs. The exclusion criteria were as follows: (1) patients had underlying congestive hepatopathy or iron-deposition liver disease, such as hereditary hemorrhagic telangiectasia, Budd–Chiari syndrome, chronic portal vein occlusion and Wilson’s disease; (2) No MR enhancement scans were performed within one month before and after clinical and (or) pathology data collection; (3) the MR protocol cannot match the requirement for the LI-RADS process; and (4) without pathological diagnosis or lack typical MR imaging features and size stability at imaging for 12 months. The process of patient selection for the study cohort is shown in Fig. 1.

### MRI examination

All patients were scanned in a supine position on a 1.5T (Magnetom Avanto, SIMENS) or a 3.0T whole-body MRI scanner (Discovery MR750, GE Healthcare) with an eight-channel phased-array torso coil centered over the abdomen. The routine contrast-enhanced liver MRI protocol included in-phase and out-of-phase, T2-weighted, diffusion-weighted (DW), unenhanced T1-weighted, dynamic, transitional-phase (TP) and hepatobiliary-phase (HBP) sequences. For dynamic phase imaging, the contrast agent gadopenate meglumine (Magnevist; Bayer Healthcare) or gadoxetic acid (Primovist; Bayer Healthcare) was injected through the elbow vein or the dorsal hand vein at a dose of 0.2 mL/kg or 0.025 mmol/kg body weight at a rate of 1–2 mL/s, followed by a 20-mL saline flush. Double-phase arterial phase (AP) images were obtained 18 s (early AP) and 28 s (late AP) after the contrast agent arrived at the thoracic artery using bolus triggering. Portal venous-phase (PVP) and delayed phase (DP) images were obtained 60 s and 4 min for gadopenate meglumine enhancement, while TP and HBP images were obtained 3 min and 15–20 min after gadoxetic acid administration, respectively. The scanning parameters are listed in Table E1.

### Image analysis

Two faculty abdominal radiologists (ZXZ, YND each with > 8 years of experience in hepatic imaging who were blinded to information on clinical history or pathology results reviewed the MRI imaging independently, and in the case of any discrepancies between the two reviewers, a third board-certified abdominal radiologist (ZBL) with 20 years of experience in hepatic imaging was enrolled to re-evaluate the imaging and obtain a final diagnosis and record the discrepancies. All of the above interpretations were based on the LI-RADS v2018 definitions shown in Table E2, including MFs and AFs (malignancy in general, HCC in particular, benignity) on MRI [6–8, 16]. The preliminary classification of the lesions was based only on MFs, such as nonperipheral arterial-phase hyperenhancement (APHE), enhancing ‘capsule’, and nonperipheral ‘washout’, and recorded by the third radiologists. Subsequently, LI-RADS categorization was assigned using MFs and independent significant AFs screened from all lesions and LR-3/4 categories in combination using the LI-RADS principle. Refer to the application rules of LI-RADS, for lesions with only malignant AFs, marked as ‘upgrade’, for lesions with only benign AFs, marked as ‘degrade’, and for lesions with both malignant and benign AFs, mark it as ‘retain’. During the interpretation process, the features causing the change in categories were recorded.

### Reference standard

For liver observations, pathologic analysis was the most recommended reference standard, including results from surgical excision or puncture biopsy as the malignancies were confirmed by. The benign lesions were enrolled mainly by typical MRI imaging features and stability in size and imaging for at least 12 months. All histological specimens were reviewed and diagnosed by an attending histopathologist and approved by a senior histopathologist with at least 10 years of experience in liver pathology blinded to all clinical data, and MRI results confirmed the histological diagnosis according to the World Health Organization (WHO) classification system [17]. In this study, enrollment methods include pathological diagnosis and inclusion based on MRI imaging characteristics. For pathological diagnosis, surgical excision and percutaneous needle biopsy were using, and for MRI imaging characteristics, stable or regressed size on follow-up and some typical imaging features were used to enroll patients.

### Statistical analysis

All analyses were performed on a per-nodule basis. The frequency of occurrence of each MF and AF was recorded and calculated using the chi-square test or Fisher’s exact test for the HCC group and non-HCC group as well as the LR-3/4/5 categories. Continuous data are summarized as the mean ± standard deviation, and categorical data are expressed as the median and interquartile range (IQR). The normal distribution of data was examined before the statistical tests. For the cases with different observations between the two radiologists, a third radiologist determined the final presence of the features for future analysis with a unified data set. The interobserver agreement of the two radiologists was calculated using the intraclass correlation coefficient (ICC), and an ICC > 0.8 indicated good agreement. Cohen’s kappa statistic was used to grade interobserver agreement of AF-HCC and AF-LR: 0.00 (no agreement), 0.01 to 0.20 (poor), 0.21 to 0.40 (fair), 0.41 to 0.60 (moderate), 0.61 to 0.80 (substantial) and 0.81 to 1.00 (nearly perfect). The McNemar test was used for the sensitivity and specificity of HCC diagnosis using the two adjustment methods of AF-HCC and AF-LR. To determine the strength of the association between HCC and non-HCC diagnosis, different categories and imaging features, the diagnostic odds ratios (DORs) and beta values of MFs and AFs were calculated using univariable and multivariable logistic regression models, with *p* < 0.10 in the analysis, and forward stepwise elimination was performed to adjust the clustering effect. The logistic regression model was also cross-verified internally with a proportion of 7:3 in the training group and validation group. The change in diagnostic performance after applying the screened AFs in the LR-3/4 categories was evaluated using McNemar’s test and Bonferroni correction to compare the sensitivity and specificity before and after the application of the AFs.

The collected data were analyzed in SPSS Version 25.0 (IBM SPSS Statistics for Windows, IBM Corp., Armonk, NY, USA) and R Version 4.2. A P value less than 0.05 was considered statistically significant.

## Results

### Patients and observations

The clinical characteristics and MRI findings of the 460 patients and 473 nodules are summarized in Table 1. A total of 311 lesions in 302 patients were included in the HCC group, including 185 cases confirmed by surgical excision and 126 cases confirmed by percutaneous needle biopsy. The non-HCC group included four types of lesions: (1) non-HCC malignancies: 5 intrahepatic cholangiocarcinoma and 1 non-Hodgkin’s lymphoma all determined by pathological diagnosis; (2) benign observations confirmed by pathology: including 14 hepatic focal nodular hyperplasia (FNH), 6 inflammatory pseudotumors of the liver (IPL), 6 high-grade dysplastic nodules (HGDN), 3 low-grade dysplastic nodules (LGDN) and 3 regenerative nodules (RN) determined by surgical excision and 6 IPL by puncture biopsy; (3) stable or regressed size on follow-up: 36 lesions in 35 patients; and (4) typical imaging features: 82 lesions in 79 patients were enrolled. 138 patients were treated with liver specific contrast agents, among them, 98 patients were in the HCC group and 40 patents were in the non-HCC group, accounting for 32.45% (98/302) and 25.31% (40/158), respectively. 322 patients were treated with liver non-specific contrast agents. 204 patients were in the HCC group and 118 patients were in the non-HCC group, accounting for 67.55% (204/302) and 74.68% (118/158), respectively. Among all enrolled patients, the proportion of male patients was significantly higher than that of female patients in the HCC group. The median age of all the patients was 54.24 ± 11.32 years, with no statistic difference among each group. In addition, BMI (*P* = 0.034), cirrhosis (*P* = 0.00), and some laboratory indexes, such as ALT (*P* = 0.044), TBIL (*P* = 0.000), and PLT (*P* = 0.004), indicating liver function estimation and AFP (*P* = 0.000), were significantly difference between the HCC group and the non-HCC group. Patients who were assessed to Child‒Pugh class A accounted for the most patients, with proportions of 81.8% and 88.0%, respectively. Among all the enrolled pathologically determined HCC lesions, moderately differentiated nodules (51.8%) were more common than well differentiated (17.4%) and low differentiated nodules (30.9%).

**Table 1:** Clinicopathologic characteristics of patients and hepatic observations

Characteristic	Total	HCC group	Non-HCC group	P -value
**Patient** (*n* = 460)	460	302	158	
Mean age (years) *	54.24 ± 11.32	56.05 ± 9.65	50.75 ± 13.31	0.061
Sex				0.000
Male	347 (75.4%)	257 (85.1%)	90 (57.0%)	
Female	113 (24.6%)	45 (14.9%)	68 (43.0%)	
BMI	23.6 (21.3–25.4)	23.3 (20.9–24.4)	23.7 (21.8–26.0)	0.034
Cirrhosis				0.000
Presence	280 (60.9%)	191(63.2%)	89 (56.3%)	
Absence	180 (39.1%)	111 (36.8%)	69 (43.7%)	
Cause of liver disease				0.213
Hepatitis B virus	368 (78.0%)	253 (81.6%)	115 (71.0%)	
Hepatitis C virus	62 (13.1%)	37 (11.9%)	25 (15.4%)	
Alcohol	29 (6.1%)	11 (3.5%)	18 (11.1%)	
NASH	13 (2.8%)	9 (2.9%)	4 (2.5%)	
Child–Pugh Class				0.000
Class A	386 (83.9%)	247 (81.8%)	139 (88.0%)	
Class B	60 (13.0%)	42 (13.9%)	18 (11.4%)	
Class C	14 (3.0%)	13 (4.3%)	1 (0.6%)	
Hepatic function index**				
AST	20.4 (29–50.7)	29.3 (20.9–52)	28.3 (18.5–40.1)	0.188
ALT	29.6 (21.9–47.9)	30.3 (23-48.6)	28.1 (20.6–46.8)	0.044
TBIL (µmol/L)	13.6 (9.9–20.2)	14.7 (11.1–20.5)	12.1 (8.8–17.5)	0.000
PT	12.3 (11.6–13.5)	12.3 (11.7–13.5)	12.2 (11.4–13.6)	0.161
PLT	143.7 (95.0–194.5)	140.0(91.0–183.0)	152.5 (111.8-218.7)	0.007
AFP**	5.83(2.7–43.1)	13.1(3.3–143.8)	3.1(1.9–5.98)	0.000
**Lesions**(*n* = 473)	473	311	162	
No. of nodules				0.317
1	447 (94.5%)	293(94.2%)	154 (95.1%)	
2	13 (6.6%)	9 (2.9%)	4 (2.5%)	
Mean size (cm)	2.91 ± 2.33	3.34 ± 1.75	2.08 ± 1.75	0.000
Degree of differentiation of HCC				
Well differentiated		54 (17.4%)		
Moderately differentiated		161 (51.8%)		
Low-differentiated		96 (30.9%)		
Standard reference of diagnosis				0.000
Pathologic diagnosis	355 (75.1%)	311 (100%)	44 (27.2%)	
Typical imaging features with size stability	118 (24.9%)	0 (0%)	118 (76.6%)	

1-**Data are shown as the median (IQR); *Data are reported as the mean ± standard deviation

2-HCC: cholangiocarcinoma, IQR: interquartile range, AST: aspartate aminotransferase ALT: alanine aminotransferase TBIL: total bilirubin, PT: prothrombin time PLT: platelet AFP: alpha fetoprotein

### Frequency of major and ancillary features

The frequencies of MFs and AFs of LI-RADS *v*2018 in the HCC group, non-HCC group and different categories of groups HCC group and non-HCC group as well as in different categories were observed and recorded, as shown in Table 2. When only relying on MFs for classification in the HCC group, the lesions classified to LR-3 was 44, classified to LR-4 was 89 and LR-5 was 178. In the Non-HCC group, the number of lesions classified for LR-3, LR-4 and LR-5 were 109, 40,13, respectively. Since the tumor growth follow-up period specified by LI-RADS *v*2018 was 24 months, the observation period for follow-up lesions in this study was only 12 months, which cannot meet the follow-up requirements of LI-RADS. To ensure the accuracy of the results, in this study, “threshold growth”, “dimensional stability ≥ 2y” and “size reduction” were excluded from the calculation. Additionally, the current LI-RADS version (v. 2018) has been simplified and does not require the lesion to be visualized before the CT or MRI study by ultrasound [18]. The frequency of MFs and tumor diameters were significantly different among all the groups (*P* = 0.000). Regarding AFs favoring malignancy in general, the frequency of mild-moderate T2 hyperintensity and restricted diffusion was significantly higher in the HCC group than in the non-HCC group. Corona enhancement was more common in the LR-4 and LR-5 categories than in the LR-3 category (LR-3 vs. LR-4, *P* = 0.017; LR-3 vs. LR-5, *P* = 0.015; and LR-4 vs. LR 5, *P* = 0.145) but was also significantly more common in the HCC group than in the non-HCC group. Among AFs favoring HCC in particular, except for nodule-in-nodule appearance (HCC vs. non-HCC, *P* = 0.554; LR-3 vs. LR-4, *P* = 0.464; LR-3 vs. LR-5, *P* = 0.299; and LR-4 vs. LR-5, *P* = 0.231), the frequencies of all the other features were significantly different among all the groups. The number of AFs favoring benignity was small in each subgroup, especially in the HCC group and LR-5 group, and the frequency was less than or equal to 5. Regarding agreement between the two radiologists, the ICC was in the range of 0.824–0.871 for the HCC/non-HCC group and 0.814–0.852 in the LR-3/4/5 group.

**Table 2:** Comparison of imaging features of HCCs and non-malignant nodules, LR-3 and LR-4

LI-RADS features	HCCs(*n* = 311)	Non-HCC nodules(*n* = 162)	P value	LR-3 lesions(*n* = 153)	LR-4 lesions(*n* = 129)	LR-5 lesions(*n* = 191)	P value**
**Major HCC features**							
Diameter: <10 mm	4.2(13/311)	25.3(41/162)	0.000	31.4(48/153)	3.9(5/129)	0.5(1/191)	0.000
Diameter: 10–19 mm	25.1(78/311)	42.0(68/162)	0.000	60.8(93/153)	17.8(23/129)	15.7(30/191)	0.000
Diameter:≥20 mm	70.7(220/311)	32.7(53/162)	0.000	7.8(12/153)	78.3(101/129)	83.8(160/191)	0.000
Non-rim APHE	79.7(248/311)	55.6(90/162)	0.000	52.3(80/153	53.5(69/129)	99.0(189/191)	0.000
Non-peripheral washout	59.8(186/311)	10.5(17/162)	0.000	7.2(11/153)	41.1(53/129)	72.8(139/191)	0.000
Enhancing capsule	56.9(177/311)	11.1(18/162)	0.000	1.3(2/153)	41.1(53/129)	73.3(140/191)	0.000
**Ancillary features Favoring malignancy in general**							
Mild-moderate T2 hyperintensity	81.0(252/311)	50.0(81/162)	0.000	52.9(81/153)	72.9(94/129)	82.7(158/191)	0.000
Fat sparing in solid mass	9.0(28/311)	9.9(16/162)	0.742	7.8(12/153)	12.4(16/129)	8.4(16/191)	0.359;0.377;0.931
Iron sparing in solid mass	4.8(15/311)	0.6(1/162)	0.015	1.3(2/153)	4.7(6/129)	4.2(8/191)	0.220;0.170;0.158
Corona enhancement	10.3(32/311)	9.9(16/162)	1.000	5.2(8/153)	15.5(20/129)	10.5(20/191)	0.017;0.015;0.145
TP hypointensity	25.4(79/311)	13.0(21/162)	0.002	16.3(25/153)	25.6(33/129)	22.0(42/191)	0.155;0.151;0.235
HBP hypointensity	25.7(80/311)	14.8(24/162)	0.007	19.6(30/153)	25.6(33/129)	21.5(41/191)	0.471;0.476;0.730
Restricted diffusion	87.1(271/311)	58.6(95/162)	0.000	60.1(92/153)	82.9(107/129)	88.0(168/191)	0.000
**Ancillary features Favoring HCC in particular**							
Nodule-in-nodule appearance	1.0(3/311)	0(0/162)	0.554	0(0/153)	0.8(1/129)	1.0(2/191)	0.464;0.299;0.231
Mosaic appearance	11.6(36/311)	0(0/162)	0.000	0(0/153)	10.1(13/129)	12.0(23/191)	0.000
Blood product in mass	17.7(55/311)	0(0/162)	0.000	1.3(2/153)	16.3(21/129)	16.8(32/191)	0.000
Fat in mass	30.5(95/311)	11.1(18/162)	0.000	15.0(23/153)	20.2(26/129)	33.5(64/191)	0.000
Non-enhancing capsule	13.5(42/311)	3.7(6/162)	0.001	3.9(6/153)	10.1(13/129)	15.2(29/191)	0.003;0.002;0.001
**Ancillary features Favoring benignity**							
Parallel blood pool	1.0(3/311)	9.3(15/162)	0.000	7.2(11/153)	5.4(7/129)	0(0/191)	0.001
Undistorted vessels	3.5(11/311)	0.6(1/162)	0.066	0.7(1/153)	3.9(5/129)	3.1(6/191)	0.182;0.124;0.165
Iron in mass more than liver	0(0/311)	0(0/162)	NA	0(0/153)	0(0/129)	0(0/191)	NA
Marked T2 hyperintensity	1.0(3/311)	21.6(35/162)	0.000	16.3(25/153)	8.5(11/129)	1.0(2/191)	0.000
HBP isointensity	1.6(5/311)	16.7(27/162)	0.000	15.0(23/153)	5.4(7/129)	1.0(2/191)	0.000

**: 1.P value was 0.000: there was statistical difference among the three groups of LR-3/LR-4/LR-5, and the p values were all 0.000

2. P value was specific number: there was no statistical difference among the three groups of LR-3/LR-4/LR-5. The three values were LR3 vs. LR-4; LR-3 vs. LR-5; LR-4 vs. LR-5, respectively

### Logistic regression model for screening features and model self-validation

**Fig. 2:**
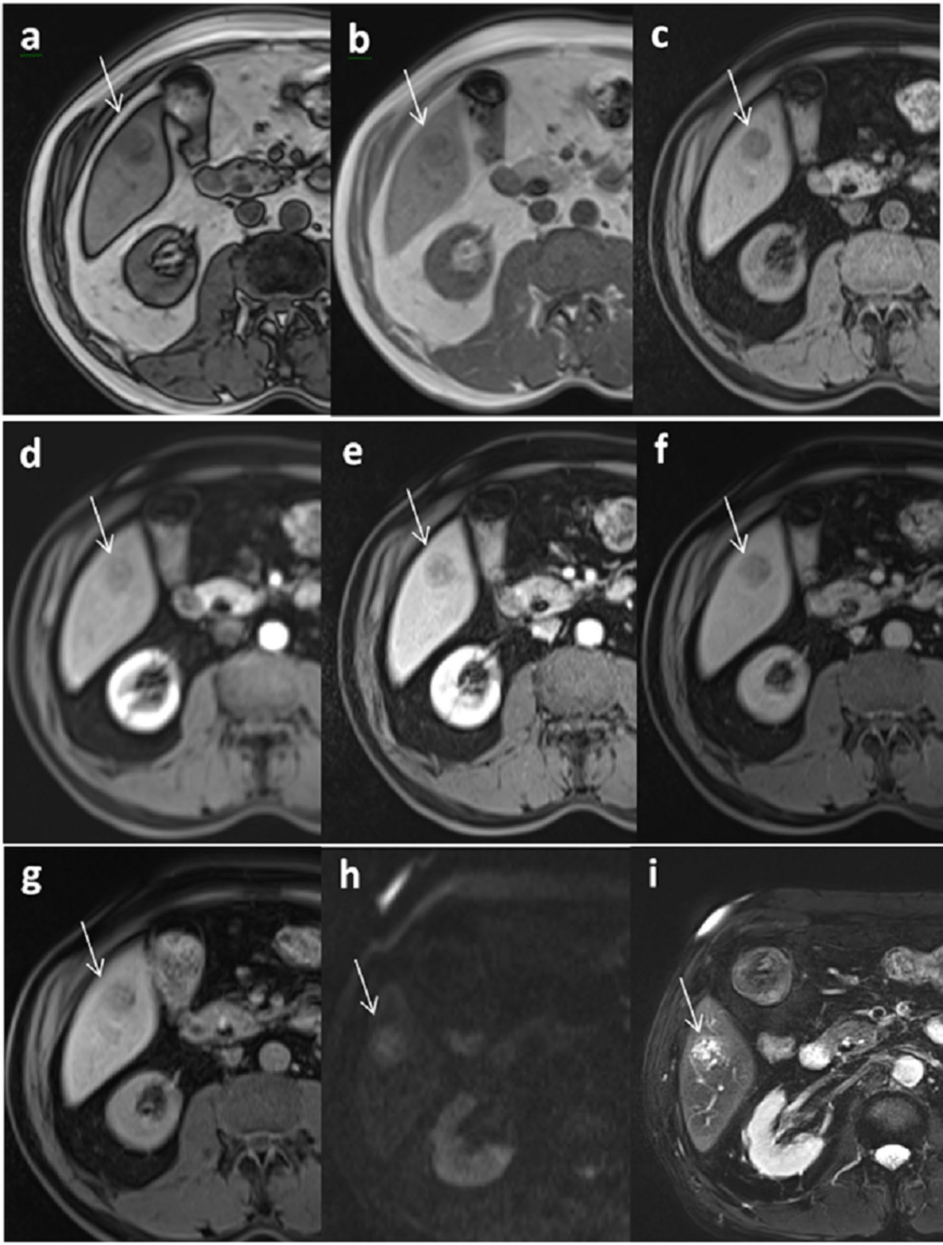
A male patient with chronic hepatitis B and a surgically confirmed hepatocellular carcinoma. **a**, **b** showed that the signal in the out-phase of the lesion reduced compared with that in the in-phase, indicating that the lesion contains fat. T1WI shows hypointensity (**c**), no obvious enhancement in the late arterial phase (AP) (**d**), and the enhancement degree in the portal venous phase (PVP) was still hypointensity (**e**), TP phase (**f**) and HBP phase (**g**) were hypointensity, and both T2WI (**i**) and DWI (**h**) showed hyperintensity. The nodule was 1.5 cm and was classified into LR-4 basing on the major features. Whenusing AF-HCC to classify the nodule, it can be upgraded, especially for the feature of TP hypointensity. This nodule was confirmed as hepatocellular carcinoma after surgical resection

**Fig. 3:**
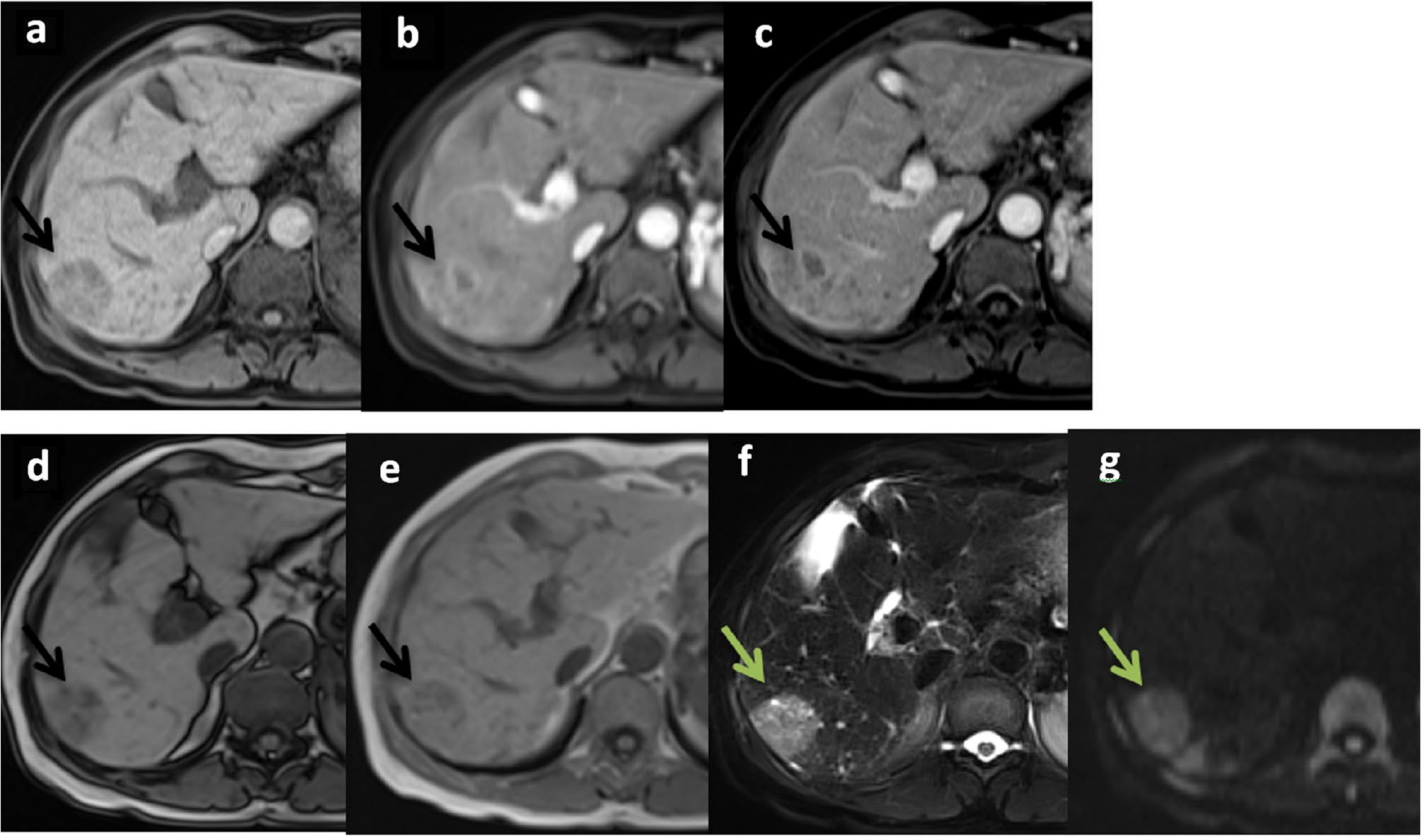
Histologically proven hepatocellular carcinoma (HCC) in a male patient with hepatitis B virus–related liver cirrhosis. Axial images of 1.5-T enhanced MRI (**a**–**g**) showed a lesion (arrows) in segment VI and segment VII junctional zone of the liver with a diameter of 3.2 cm, showing T1-weighted hypointensity (**a**), and without certain APHE in arterial phase (AP) (**b**), in portal venous phase (PVP) (**c**) showing hypointensity, the signals in out-phase reduced than that in in-phase (**d**-**e**), both T2WI (**f**) and DWI (**g**) showed hyperintensity. Based on major HCC features, this nodule would be assigned as LR-4 because of the lack of APHE. However, based on AF-LR, it can be upgraded

The results of univariate and multivariate analysis showed that the AFs supporting malignant tumors with high diagnostic ability that were beneficial to predicting HCC and LR-5 lesions were screened out in the HCC/non-HCC group (AF-HCC) and in different category groups (AF-LR), respectively *(*Tables 3 and 4*)*. The malignant AF-HCCs were mild–moderate T2 hyperintensity, restricted diffusion, TP hypointensity (*P* = 0.005, < 0.001, = 0.032) (Fig. 2*)*, and AF-LR were restricted diffusion, mosaic architecture, and fat in mass (*P* < 0.001, = 0.020, = 0.036) (Fig. 3*)*, as shown in Tables 3 and 4. The benign AF-HCC and AF-LR were consistent, with both marked T2 hyperintensity and HBP isointensity (HCC/non-HCC group: *P* < 0.001, = 0.013, LR-3/4 group: *P* < 0.001, = 0.016).

**Fig. 4:**
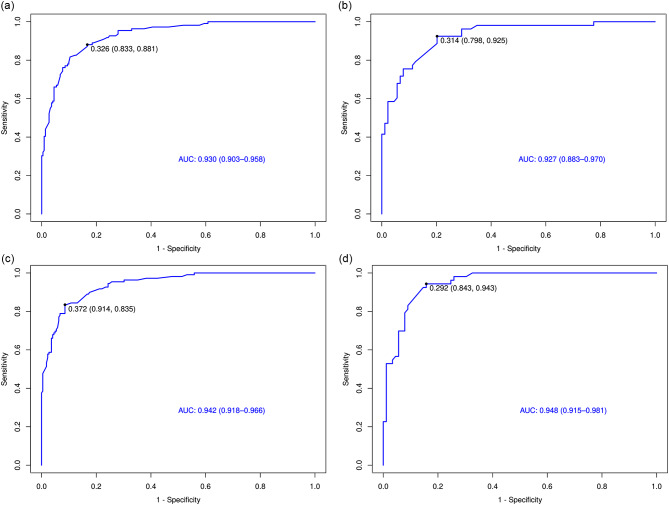
ROC curve of test group and verified group for LR3/4 (**a**, **b**) and all lesions (**c**, **d**)

At the same time, we performed internal cross-validation in the logistic regression model of the HCC/non-HCC group and LR-3/LR-4 group to verify the efficiency of the model. The training group and validation group were divided by random stratified sampling of 7:3, and then the ROC curve (Fig. 4), decision curve (*Fig*. E1) and calibration curve (*Fig*.E2) were generated. The area under the curve (AUC) of the two sets of logistic regression models in the training group and validation group was 0.948 (95% CI: 0.915–0.981) and 0.930 (95% CI: 0.903–0.958) for the HCC/non-HCC groups and 0.942 (95% CI: 0.918–0.966) and 0.930 (95% CI: 0.883–0.970) for the LR-3/4 groups, respectively.

**Table 3:** Logistic regression analyses of ancillary features in LI-RADS v2018 for all lesions

Variables	Univariable analysis			Multivariable analysis		
	Beta	OR (95%CI)	P	aBeta	aOR (95%CI)	aP
Nodule-in-nodule architecture	-1.40	0.25 (0.10–0.59)	0.002	-1.34	0.26 (0.06–1.13)	0.073
Nonenhancing “capsule”	-13.92	0.00 (0.00 - Inf)	0.978			
Mosaic architecture	-17.04	0.00 (0.00 - Inf)	0.979			
Blood products in mass	-17.11	0.00 (0.00 - Inf)	0.974			
Fat in mass	0.83	2.29 (1.35–3.86)	0.002			
Subthreshold growth	14.22	1505228.15 (0.00 - Inf)	0.979			
Restricted diffusion	1.59	4.92 (3.11–7.78)	< 0.001	1.81	6.11 (2.56–14.61)	< 0.001
Mild–moderate T2 hyperintensity	-0.05	0.96 (0.51–1.80)	0.888	1.57	4.79 (1.62–14.14)	0.005
Coronal enhancement	1.45	4.27 (2.81–6.49)	< 0.001			
Fat sparing	0.10	1.11 (0.58–2.11)	0.756			
Iron sparing	-2.10	0.12 (0.02–0.94)	0.043	-2.03	0.13 (0.01–1.18)	0.070
Transitional-phase hypointensity	-1.26	0.28 (0.16–0.49)	< 0.001	-0.95	0.39 (0.16–0.92)	0.032
Hepatobiliary-phase hypointensity	0.69	1.99 (1.20–3.29)	0.007			
Parallels blood pool enhancement	2.35	10.48 (2.99–36.75)	< 0.001			
Undistorted vessels	-1.78	0.17 (0.02–1.32)	0.090	-1.74	0.18 (0.02–1.70)	0.133
Marked T2 hyperintensity	3.34	28.29 (8.55–93.66)	< 0.001	2.96	19.22 (3.38–109.24)	< 0.001
HBP isointensity	2.50	12.24 (4.61–32.47)	< 0.001	1.72	5.57 (1.44–21.56)	0.013

**Table 4:** Logistic regression analyses of ancillary features in LI-RADS v2018 for LR3/LR4 observations

Variables	Univariable analysis			Multivariable analysis		
	Beta	OR (95%CI)	P	aBeta	aOR (95%CI)	aP
Nodule-in-nodule architecture	-16.78	0.00 (0.00 - Inf)	0.980	-16.81	0.00 (0.00 - Inf)	0.994
Nonenhancing “capsule”	-14.69	0.00 (0.00 - Inf)	0.987			
Mosaic architecture	-1.53	0.22 (0.07–0.67)	0.038	-2.34	0.10 (0.01–0.69)	0.02
Blood products in mass	-17.87	0.00 (0.00 - Inf)	0.983			
Fat in mass	-1.53	0.22 (0.07–0.67)	0.008	-2.42	0.09 (0.01–0.86)	0.036
Subthreshold growth	14.46	1903499.57 (0.00 - Inf)	0.987			
Restricted diffusion	1.51	4.52 (2.52–8.10)	< 0.001	1.89	6.64 (2.70–16.30)	< 0.001
Mild–moderate T2 hyperintensity	1.44	4.23 (2.50–7.17)	< 0.001	0.18	1.19 (0.49–2.88)	0.695
Coronal enhancement	-0.61	0.54 (0.24–1.21)	0.135			
Fat sparing	0.19	1.21 (0.55–2.67)	0.631			
Iron sparing	-2.11	0.12 (0.01–1.00)	0.050	-2.04	0.13 (0.01–1.36)	0.089
Transitional-phase hypointensity	-1.04	0.35 (0.19–0.65)	< 0.001	-0.88	0.42 (0.06–3.10)	0.393
Hepatobiliary-phase hypointensity	-0.94	0.39 (0.22–0.70)	0.001	0.00	1.00 (0.14–7.13)	1.000
Parallels blood pool enhancement	1.58	4.85 (1.37–17.15)	0.014	0.57	1.76 (0.31–9.97)	0.521
Undistorted vessels	-1.75	0.17 (0.02–1.50)	0.111			
Marked T2 hyperintensity	2.96	19.37 (4.55–82.38)	< 0.001	3.42	30.44 (4.52–205.11)	< 0.001
HBP isointensity	2.26	9.59 (2.84–32.42)	< 0.001	1.73	5.62 (1.39–22.73)	0.016

### Diagnostic performance of criteria using screened ancillary features

According to the rules of LI-RADS, well-performing AF-HCC and AF-LR were reapplied to LR-3 and LR-4 observations. The diagnostic manifestations for HCC based on the adjusted classification are shown in Table 5. In the HCC group, 85% (AF-HCC) and 85.6% (AF-LR) of the lesions showed upgrade changes, and only 1 lesion showed degradation after the use of features screened from the LR-3/4 lesions. In the Non-HCC group, 40.9% (AF-HCC) and 47% (AF-LR) of lesions were upgraded, 10.7% (AF-HCC) and 9.4% (AF-LR) of lesions were degraded, and 48.3% (AF-HCC) and 43.6% (AF-LR) retained the grade. Restricted diffusion in AF-LR can lead to the upgrading of 83.9% (26/31) of HCC lesions, the mosaic appearance and fat in mass content make the remaining HCC lesions (5/31) complete the grade upgrade. Using AF-HCC, restricted diffusion and mild-moderate T2 hyperintensity appear during grade adjustment (23 lesions and 22 lesions, respectively). TP hypointensity alone improved the classification of 2 lesions. After the above grades changed, the diagnostic sensitivity for HCC were 84.96% using AF-HCC and 85.71% using AF-LR, the specificity were 89.26% using AF-HCC and 90.60% using AF-LR, which made a significant difference (*P* = 0.000). And the kappa value for the two methods of AF-HCC and AF–LR were 0.695, reaching a substantial agreement.

**Table 5:** The diagnostic manifestations for LR-3/LR-4 lesions in HCC group and Non-HCC group base on AF-HCC and AF-LR

Variable	level	Overall	HCC group (LR-3/4 *n* = 133)	Non-HCC group (LR-3/4 *n* = 149)	Sensitivity (%)	Specificity (%)	Kappa test	P
**(a) Based on ancillary features screened from all lesions**							0.695	0.000
	Degrade	5.7(16/282)	0.0 (0/133)	10.7(16/149)	84.96 (113/133)	89.26 (133/149)		
	Retain	32.6(92/282)	15.0(20/133)	48.3(72/149)				
	Upgrade	61.7(174/282)	85.0(113/133)	40.9(61/149)				
**(b) Based on ancillary features screened from LR-3/LR-4 lesions**								
	Degrade	5.3(15/282)	0.8(1/133)	9.4(14/149)	85.71 (114/133)	90.60 (135/149)		
	Retain	29.4(83/282)	13.5(18/133)	43.6(65/149)				
	Upgrade	65.2(184/282)	85.6(114/133)	47.0(70/149)				

## Discussion

In previous LI-RADS-related studies, researchers were interested in screening out AFs to replace MFs and use AFs to improve the classification of small lesions or LR-3/4 lesions [15, 19–23]. However, most of these studies screened features based on all lesions, which may have overlooked the impact of the frequency and diagnostic ability of AFs in LR-5 lesions on the results. In this study, a total of 311 cases of pathologically confirmed HCC and 162 cases of non-HCC nodules were included, and 96.3% (156/162) of the latter were benign nodules. After classification according to the MFs of LI-RADS, 191 nodules were classified into the LR-5 category, accounting for 61.4% (191/311) and 40.4% (191/473) of HCC lesions and all lesions, respectively. In contrast, LR-3 and LR-4 nodules accounted for approximately 59.6% (282/473) of the total number of lesions. The goal of the LI-RADS diagnostic algorithm is to provide 100% specificity for the diagnosis of HCC because the definitive diagnosis of HCC is typically based on imaging, while histologic confirmation is not needed prior to treatment, which is different from most other malignancies [9]. Therefore, the application rules of LI-RADS prevent the adjustment of the LR-4 lesion to the LR-5 category, and few LR-5 lesions could reach downgrade (from LR-5 to LR-4) [8, 24], which causes the AFs of LR-5 lesions to be overlooked. Therefore, we tried to ignore the impact of LR-5 AFs on the results and only included AFs of LR-3 and LR-4 categories in different category groups for calculation and compared them with those screened based on all lesions. After logistic regression, it was found that the AFs supporting malignancy were not exactly the same. In the HCC/non-HCC group, the screened AFs were mild–moderate T2 hyperintensity, restricted diffusion, and TP hypointensity, which was consistent with some previous literature [23, 25], and in the different category groups, restricted diffusion, mosaic architecture, and fat in mass were screened out. The benign AFs screened out in the two groups were the same, and both had marked T2 hyperintensity and HBP isointensity. At the same time, to verify the diagnostic performance of the established logistic regression model, we conducted random sampling internal cross-validation on both groups of models and drew the ROC curves, calibration curves and decision curves of the training set and validation set of the two groups. The areas under the curve for the two groups reached 0.948 and 0.930 for the HCC/non-HCC groups and 0.942 and 0.930 for the LR-3/4 groups, confirming the high standards and repetitiveness of the logical regression model.

The diagnosis and treatment of LR-3 lesions in LI-RADS requires regular imaging follow-up, although other guidelines (e.g., Asia Pacific Association for the Study of the Liver [APASL] or Korean Liver Cancer Association-National Cancer Center [KLCANCC] guidelines) include most of the LR-4 observations considered clear HCC [12, 14], but in LI-RADS, it is still required to conduct a joint discussion for LR-4 lesions to determine whether to perform immediate surgery, interventional treatment or follow-up imaging. Since the two types of lesions and LR-5 lesions are treated differently, differential diagnosis and improving diagnostic sensitivity and specificity as much as possible become key. Prior to this, some studies reset the LI-RADS rules, applied the screened AFs to the LR-4 category and classified them into the m-LR-4 category. It was found that this adjustment increased the sensitivity of HCC diagnosis (35.6 − 88.5%) but reduced specificity (86.2% − 75.9%) [25], which was similar to our results after applying AF-HCC and AF-LR to category adjustment. In previous studies, the diagnostic specificity of lesions classified into LR-3/4 based on MFs reached 81% (LR-3 category) and 87% (LR-4 category), respectively [26]. In the results of this study, after the grades changed using AF-HCC and AF-LR, the diagnostic sensitivity for HCC were 84.96% and 85.71%, and the specificity were 89.26% and 90.60% respectively, which made a significant difference consistent with previous research. Due to the difference in inclusion time, the bias of the included cases were exsist: the growth cycle of liver cirrhosis nodules classified into LR-3 developed HCC was 5.1%, 12.5%, and 14.8% patients within 6, 12, and 18 months, respectively [27]. However, the follow-up period of the observed lesions in this study was only 12 months, which may cause more regenerative nodules (RNs) or dysplastic nodules (DNs) to be included in the LR-3/4 category than in the other studies [28].When adjusting the categories of LR-3/4 categories, we found something worth noting. The frequency of AFs used to adjust categories was different with the two methods: restricted diffusion in AF-LR can lead to the upgrading of most of HCC lesions, while the mosaic appearance and fat in mass content make the remaining HCC lesions complete the grade upgrade. This is different from AF-HCC, where restricted diffusion and mild-moderate T2 hyperintensity appear during grade adjustment (23 lesions and 22 lesions, respectively). TP hypointensity alone improved the classification of 2 lesions. In some previous studies, some larger lesions have a mosaic structure due to intralesional fatty components, necrosis, or hemorrhage at histopathology [8, 24, 29]. Similarly, the lesion contains lipids to change the degree and method of the arterial stage of the lesion, which also makes the lesion classify into the LR-3 or LR-4 categories due to the lack of nonrim APHE [9, 24], which is similar to the results of Christian B. van der Pol’s study for filtering features by machine learning [15]. In this study, when AF-HCC and AF-LR were used to adjust the categories of LR-3/LR-4 lesions, the consistency between the two methods was good, and both the sensitivity and specificity for AF-HCC is higher than that for AF-LR, which makes a significant difference.

This study has several limitations: First, we retrospectively included hepatic nodules confirmed by histologic assessment in HCC group and both histologic assessment and MR imaging in non-HCC group, which inevitably resulted in verification bias. Second, there is insufficient research on LR-5 AFs. We referred to the literature and believed that the AFs of the LR-5 category are similar to those of the HCC group but did not make further calculations. Third, the follow-up period for benign lesions is not consistent with LI-RADS. This defect causes us to not include the MFs and AFs related to tumor growth in the calculation, making the statistics of LI-RADS signs incomplete. Last, in order to obtain more objective results, we introduced all patients who met the inclusion and exclusion criteria into the study, including patients who received liver non-specific contrast agents and those received non-specific contrast agents, although the proportion of patients using liver-specific contrast agents is comparable in HCC group and the non-HCC group, there was no in-depth analysis of the MR characteristics of patients with specific contrast agents. We will conduct in-depth research and calculation on the above shortcomings in subsequent research.

In conclusion, this study counted the frequency and diagnostic performance of AFs in different groups and different LI-RADS category observations, screened out AFs with high diagnostic ability for HCC from the HCC/Non-HCC group and the LR-3/LR-4 group, and then reapplied it to LR-3 and LR-4 observations, determining that compared to AFs screened from all the HCC and non HCC lesions, AFs screened from only LR-3/4 leisons had better diagnostic ability in optimizing LI-RADS *v*2018 and distinguishing solid lesions of the liver.

### Electronic supplementary material

Below is the link to the electronic supplementary material.

Supplementary Material 1

Supplementary Material 2

Supplementary Material 3

Supplementary Material 4

Supplementary Material 5

Supplementary Material 6

## Data Availability

No datasets were generated or analysed during the current study.
