# Supplementary figures and images for: Optimizing LI-RADS: ancillary features screened from LR-3/4 categories can improve the diagnosis of HCC on MRI
Source: BMC Gastroenterol. 2024 Mar 21;24:117. doi: 10.1186/s12876-024-03201-2 (PMC10956370; doi:10.1186/s12876-024-03201-2)

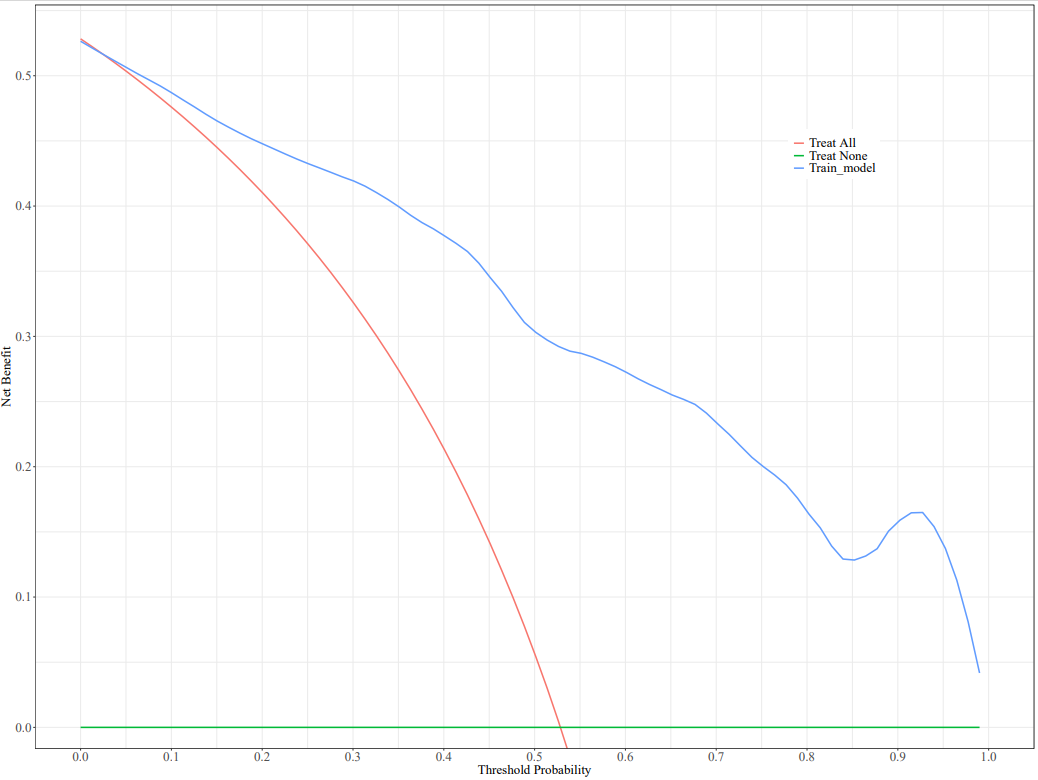

Supplement: Supplementary file 1 — Supplementary Material 1 [file 12876_2024_3201_MOESM1_ESM.png]

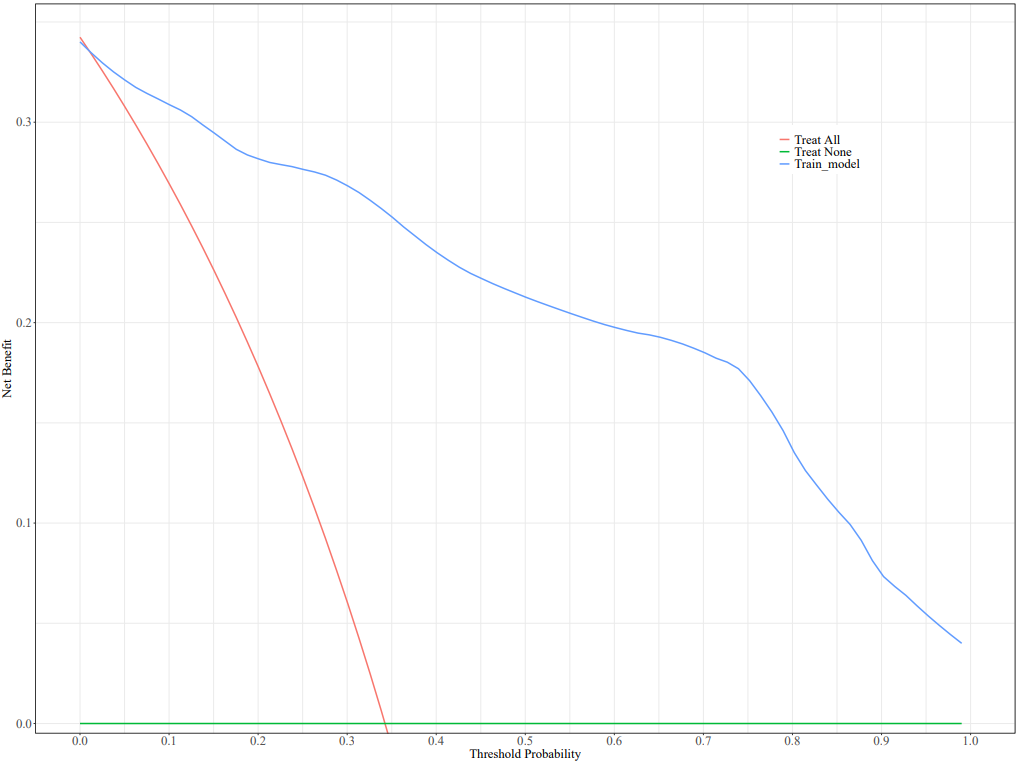

Supplement: Supplementary file 2 — Supplementary Material 2 [file 12876_2024_3201_MOESM2_ESM.png]

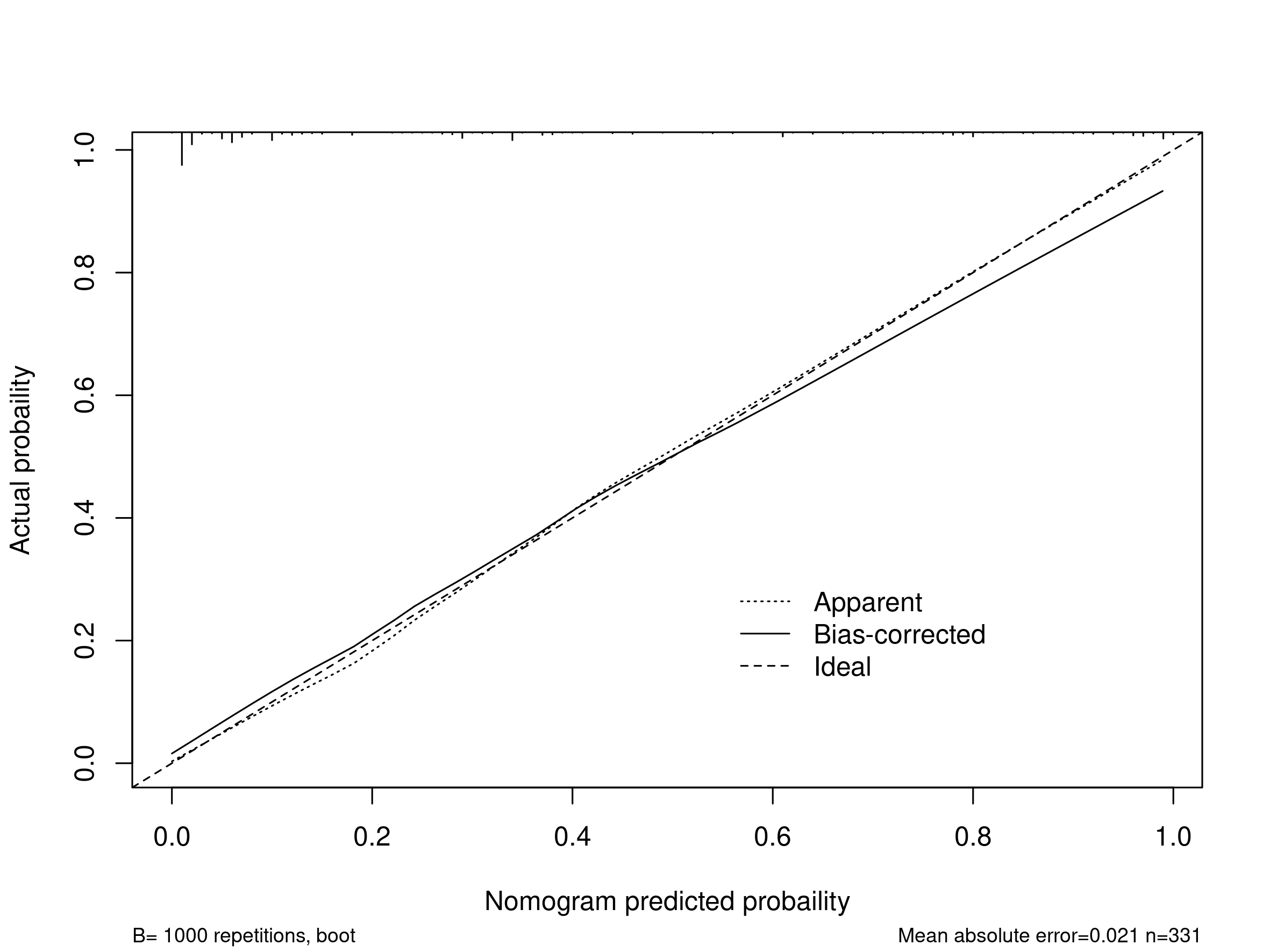

Supplement: Supplementary file 3 — Supplementary Material 3 [file 12876_2024_3201_MOESM3_ESM.png]

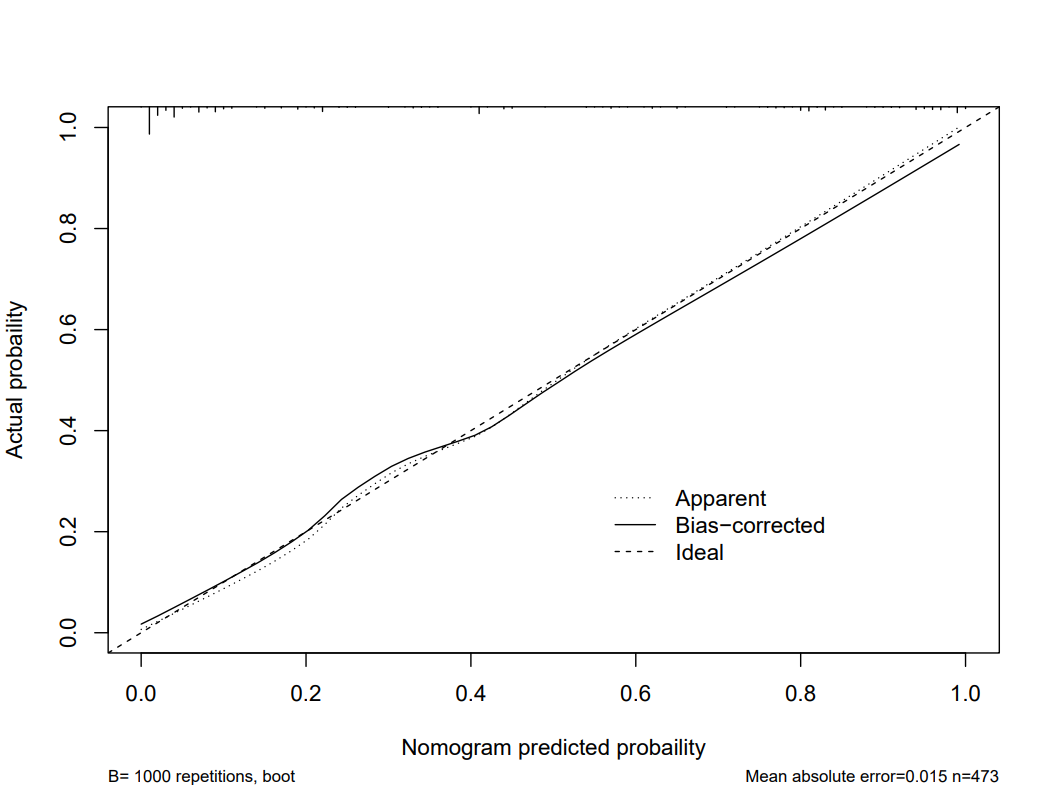

Supplement: Supplementary file 4 — Supplementary Material 4 [file 12876_2024_3201_MOESM4_ESM.png]
