# Supplementary material for: Optimizing LI-RADS: ancillary features screened from LR-3/4 categories can improve the diagnosis of HCC on MRI
Source: BMC Gastroenterol. 2024 Mar 21;24:117. doi: 10.1186/s12876-024-03201-2 (PMC10956370; doi:10.1186/s12876-024-03201-2)
Supplement: Supplementary file 5 — Supplementary Material 5 [file 12876_2024_3201_MOESM5_ESM.docx]

**Supplementary figure legends**

Figure E1: Decision curve of LR3/4 (a) and all lesions (b).

Figure E2: Calibration curve of LR3/4 (a) and all lesions (b).
