# Supplementary material for: Optimizing LI-RADS: ancillary features screened from LR-3/4 categories can improve the diagnosis of HCC on MRI
Source: BMC Gastroenterol. 2024 Mar 21;24:117. doi: 10.1186/s12876-024-03201-2 (PMC10956370; doi:10.1186/s12876-024-03201-2)
Supplement: Supplementary file 6 — Supplementary Material 6 [file 12876_2024_3201_MOESM6_ESM.docx]

Table E1: MRI parameters

|  | Field strength  (T) | Repetition time (msec) | echo time (msec) | Flip angle (degrees) | Slice thickness (mm) | Intersection gap (mm) | Band-with | Fat Suppression | Matrix | FOV (mm) | Acquisition time |
| --- | --- | --- | --- | --- | --- | --- | --- | --- | --- | --- | --- |
| T2WI (AX) | 3.0 | 11250 | 87 | 90 | 7 | 7 | 83.33 | YES | 288*288 | 38 *38 | 2min 15s |
|  | 1.5 | 5400 | 67 | 90 | 6 | 6 | 83.33 | YES | 288*288 | 38 *38 | 2min |
| T2WI (COR) | 3.0 | 1381 | 71.3 | 90 | 7 | 7 | 31.25 | NO | 320*320 | 44*44 | 28 s |
|  | 1.5 | 1200 | 70 | 90 | 5 | 5 | 31.25 | NO | 320*320 | 44*44 | 32 s |
| DWI | 3.0 | 11250 | 66.5 | 180 | 6 | 6 | 250 | YES | 128*128 | 40*36 | 2min 1s |
|  | 1.5 | 5400 | 69 | 180 | 6 | 6 | 250 | YES | 128*128 | 40*36 | 2min 2s |
| T1WI | 3.0 | 3.7 | 1.7 | 12 | 4.4 | 5 | 62.5 | YES | 256*192 | 40*36 | 10s |
|  | 1.5 | 4.4 | 2.7 | 12 | 5 | 5 | 62.5 | YES | 256*200 | 40*36 | 12s |
| LAVA | 3.0 | 3.7 | 1.7 | 12 | 4.4 | 3 | 142.86 | YES | 256*200 | 40*36 | 15min |
|  | 1.5 | 3.5 | 1.3 | 11 | 3.0 | 3.0 | 142.86 | YES | 256*200 | 40*36 | 16min |
| in-phase and out-of-phase | 3.0 | 5 | 2.4 | 70 | 6 | 6 | 128.5 | NO | 256*200 | 40*36 | 30s |
|  | 1.5 | 102 | 22 | 70 | 6 | 6 | 128.5 | NO | 256*200 | 40*36 | 28s |

Table E2: LI-RADS Version 2018 Major Features and Ancillary Features at MRI

| **Major features** | | APHE | Nonrim-like enhancement in arterial phase unequivocally greater in whole or in part than liver. Enhancing part must be higher in intensity than liver in arterial phase |
| --- | --- | --- | --- |
|  |  | “Washout” | Nonperipheral visually assessed temporal reduction in enhancement in whole or in part relative to composite liver tissue on portal venous phase |
|  |  | Enhancing “capsule” | Smooth, uniform, sharp border around most or all of an observation, unequivocally thicker or more conspicuous than fibrotic tissue around background nodules, and visible as an enhancing rim in PVP or TP |
|  |  | Size | Largest outer-edge-to-outer-edge dimension of an observation |
| **Ancillary Features** | Malignancy in general | Subthreshold growth | Unequivocal growth of a mass, less than threshold growth |
|  |  | Corona enhancement | Periobservation enhancement in late arterial phase or early PVP attributable to venous drainage from tumor |
|  |  | Fat sparing in solid mass | Paucity of fat in solid mass relative to steatotic liver or in inner nodule relative to steatotic outer nodule |
|  |  | Restricted diffusion | Signal intensity at DWI, not attributable solely to T2WI shine-through, unequivocally higher than in liver and/or ADC unequivocally lower than in liver |
|  |  | Mild-moderate T2 hyperintensity | Signal intensity at T2WI mildly or moderately higher than in liver and similar to or less than in non–iron-overloaded spleen |
|  |  | Iron sparing in solid mass | Paucity of iron in solid mass relative to iron-overloaded liver or in inner nodule relative to siderotic outer nodule |
|  |  | Transitional phase hypointensity | Signal intensity in transitional phase unequivocally less, in whole or in part, than in liver |
|  |  | Hepatobiliary phase (HBP) hypointensity | Signal intensity in HBP unequivocally less, in whole or in part, than in liver |
|  | HCC in particular | Nonenhancing “capsule” | Capsule appearance not detected as enhancing rim |
|  |  | Mosaic architecture | Presence of randomly distributed internal nodules or compartments, usually with different imaging features |
|  |  | Nodule-in-nodule architecture | Presence of smaller inner nodule within and having different imaging features than larger outer nodule |
|  |  | Fat in mass, more than in adjacent liver | Excess fat within mass, in whole or in part, relative to background liver |
|  |  | Blood products in mass | Intralesion or perilesion hemorrhage in absence of biopsy, trauma, or intervention |
|  | Benignity | Size stability for ≥2 y | No significant change in observation size measured at examinations ≥2 y apart in absence of treatment |
|  |  | Size reduction | Unequivocal spontaneous decrease in size over time, not attributable to artifact, measurement error, technique differences, or resorption of blood products |
|  |  | Parallels blood pool enhancement | Temporal pattern in which enhancement eventually reaches and then matches that of blood pool |
|  |  | Undistorted vessels | Vessels traversing observation without displacement, deformation, or other alteration |
|  |  | Iron in mass, more than in liver | Excess iron in mass relative to that in background liver |
|  |  | Marked T2 hyperintensity | Signal intensity on T2-weighted images markedly higher than in liver and similar to that in bile ducts and other fluid-filled structures |
|  |  | HBP isointensity | Signal intensity in HBP nearly identical to that in liver |
